# Characteristics and early clinical outcomes of key populations attending comprehensive community-based HIV care: Experiences from Nasarawa State, Nigeria

**DOI:** 10.1371/journal.pone.0209477

**Published:** 2018-12-20

**Authors:** Olujuwon Ibiloye, Tom Decroo, Nathaniel Eyona, Peter Eze, Peter Agada

**Affiliations:** 1 Lafia Integrated Sexual Health Centre, Society for Family Health, Lafia, Nigeria; 2 Clinical services, APIN Public Health Initiative, Abuja, Nigeria; 3 Institute of Tropical Medicine, Antwerpen, Belgium; 4 Research Foundation Flanders, Brussels, Belgium; KEMRI Wellcome Trust Research Programme, KENYA

## Abstract

**Background:**

Despite a call for differentiated care, there are limited data from sub-Saharan Africa on comprehensive community-based HIV care for key populations (KP), including commercial sex workers (CSW), men who have sex with men (MSM), and people who inject drugs (PWID). In Nigeria, a programme was implemented that liaised with community-based organizations and offered HIV testing, same-day ART initiation, and ART follow-up to KP. Here we characterize KP and their partners enrolled on ART. Our objective is to assess the early treatment outcomes and to estimate predictors of attrition among KP.

**Method:**

This is a retrospective cohort study of routinely collected data in a community-based HIV program for KP in Nasarawa state, Nigeria from August 2016 to November 2017. Variables of interest were socio-demographic, KP types, treatment outcomes, ART adherence, WHO stage, TB status and viral load. Summary statistics, logistic and Cox proportional hazard regression were used to describe the characteristics of KP and estimate predictors of attrition (patients either lost to follow-up (LTFU) or dead).

**Result:**

Seven hundred and ten (710) KP and their partners were enrolled into this study, 77.3% (549) of study participants were female and the median age was 30 years (IQR: 24–35). Respectively, 74.2%, 4.5%, 1.1% and 20% were FSW, MSM, PWID and their partners. Of 710 KP who started ART, 13.9% (99/710) discontinued after the first visit. After a median follow-up time of 7 months on ART 73.2% of patients were retained, 23.4% were LTFU, and 3.4% were dead. Lack of formal education (aHR 1.8; 95% CI 1.3–2.6) and unemployment (aHR 1.8; 95% CI 1.2–2.6) were significantly associated with attrition.

**Conclusion:**

Comprehensive community-based HIV care, including HIV testing and same-day ART is feasible. However, ART initiation on the same day of confirmatory HIV testing resulted in a high uptake of ART, but possibly inflated early attrition on ART. To mitigate early attrition among KP after same-day ART initiation, the psychosocial readiness of clients should be assessed better. We strongly recommend further studies to understand factors contributing to high attrition among the KP.

## Introduction

By 2020, the Joint United Nations Programme on HIV/AIDS (UNAIDS) aims for 90% of people living with HIV (PLHIV) to know their status, for 90% of those who know their status to be on antiretroviral therapy (ART), and for 90% of those on ART to have a suppressed viral load. Hence, it is expected that both HIV/AIDS related deaths and new HIV infections will be averted substantially. To avert new HIV infections, it will be key to enhance access and utilization of HIV care services and attain the UNAIDS 90-90-90 targets in both the general population and key populations (KP) [1–2].

In Nigeria, prevalence of HIV is on the decline, it dropped from 5.8% in 2001 to 3.1% in 2014 (ANC Survey report). However, KP, including commercial sex workers (CSW), men who have sex with men (MSM), people who inject drugs (PWID) and transgender continue to have a high HIV prevalence. HIV prevalence is respectively 22.9%, 19.4% and 8.6% in MSM, brothel-based female sex workers, and non-brothel-based female sex workers [3]. One Nigerian study showed that HIV prevalence was 4 to 10 times higher in MSM than in the general population [4]. Moreover, new infections disproportionally affect KP. Sex workers, MSM and PWID make up only 3.4% of the Nigerian population, yet account for around 32% of new HIV infections [5].

In Nigeria, ART coverage in KP is unknown [6–8]. KP are understudied and likely underserved resulting in a limited characterization of their HIV prevention, treatment, and care needs [7]. On the other hand, stigma, discrimination, poor nutrition, food insecurity and substance use are known predictors of poor linkage to care, retention in care, and viral suppression [9–10]. To overcome barriers to care, KP-friendly community-based approaches have been proposed, which may be more trustworthy and accessible than facility-based HIV care [11].

To increase access to ART services for KP, PEPFAR Nigeria has proposed strategies that are based on the principle of UNAID’s 90-90-90. Overall, the USAIDS’ project seeks to intensify case finding through strategically targeted testing, early commencement of ART for HIV positive KP and an adherence and support program with viral load testing to ensure community viral suppression [12]. In Nasarawa state, a comprehensive package of HIV care services is delivered within the community. Provided services include HIV testing, same-day ART initiation, and ART refill.

In Nigeria, treatment outcomes of comprehensive community-based HIV care for KP, including HIV testing and ART, are understudied. Therefore, this study aims to assess the early treatment outcomes and to estimate predictors of attrition (patients either lost to follow-up (LTFU) or dead) among KP on ART attending community-based HIV care, in Nasarawa state, Nigeria.

## Methods

### Study design

This study is a retrospective cohort study using routine programmatic data.

### General setting

Nasarawa state is located in central Nigeria. Its headquarters are in the town of Lafia.

It has an area of 27,117 km^2^ (10,470 sq mi) and a population of 2,040112 (Density-75/km2) at the 2006 census. The State has three National Senatorial Districts (South, North and West) and consists of 13 Local Government Areas. Our Study sites are situated in Lafia, Akwanga, Karu and Keffi local government areas of Nasarawa state.

In collaboration with implementing partners (funded either by PEPFAR or Global fund), the State Ministry of Health and the State Agency for the control of AIDS provide ART services to the general population through the various public health institutions in the state. Implementing partners in the state provide HIV prevention, care and treatment services in priority local government areas in the state. Despite increasing access to ART in the state, there is no national policy or health structure for KP that are living with HIV.

### Integrated sexual health centre

In Nasarawa state, a comprehensive community-based HIV care model was adopted to reach KP living with HIV and to increase access and utilization of HIV care services among the KP community in the state.

Community-based ART (CBART) was implemented through the Integrated Sexual Health Centre, that is situated at the state capital. From this centre, an outreach team of ART providers are deployed to surrounding communities to provide ART services. Outreach activities are organized in collaboration with community based organizations (CBO). Outreach teams consist of an ART clinician, STI providers, a triage nurse, a counselor, a pharmacist, and a Medical Laboratory Scientists. CBOs engage community facilitators (peer educators, HIV counselors and referral officers -who provide voluntary HIV testing and counselling- and treatment officers -who plan HIV outreach services for KP-. They all work within the community, pay home visits, and render services in outreach venues. KP testing HIV positive in the community are referred to either an outreach venue or to the health facility, depending on their preference.

In total, five community-based outreach venues are operational in Nasarawa state since the 31^st^ of July 2016. At these outreach-venues, HIV care is organized by the community facilitators (peer educators and referral officers) in collaboration with the ART outreach teams. Outreach venues were pre-determined locations, usually CBOs’ offices, primary health care facilities and hotels/guest houses, in 5 local government areas in the state.

Community-based HIV care includes HIV testing, same-day ART initiation (regardless of CD4 count), ART refill, STI care, and peer education sessions. Services are provided by community facilitators and the ART outreach team. Adopted strategies to improve HIV positivity yield include HIV snowball testing and counseling as well as partner testing; this strategy involves exploring sexual network of index cases and offering HIV test to index partners. Patients who test HIV positive are counselled and are proposed to start ART the same day. Stable patients on ART (clients that are adherent to medication/clinic appointment and have CD4 count > 500/ml on two consecutive tests, 6 months part) can benefit from drug pick-up by proxy. Drug pick-up by proxy involves ART drug dispensing through lay-men (i.e peer educators, community mobilizing officers, treatment partners) to patients on ART. In addition, STI care is provided to all KP. Patients that are lost to follow-up are tracked by phone call and home visits. A list of clinic defaulters is generated using appointment registers at the end of each scheduled outreach/clinic for immediate tracking by the community facilitators.

### Study population

All adult KP (18 years or older), living with HIV that were enrolled and initiated on ART in the community-based ART program (OSS) between the 1st of August 2016 and the 28th of February 2017, in Nasarawa state were enrolled into this study.

### Study period

Data were collected from visits that occurred between 1^st^ of August 2016 and 30^th^ of November 2017. Outcomes were defined on the 31^st^ of August 2017. The follow-up time between 31 August 2017 and 30 November 2017 allowed ascertaining if patients late for their next appointment on 31 August were truly LTFU. Patients who started ART at the end of February 2017, were 6 months on ART at the end of August 2017.

### Data collection and definition of variables

Data for all included participants were collected with a standardized data extraction template that contained participant-level data of interest. Retrospective data were extracted from the Health Management Information Systems (HMIS) tools. All the data used in the study were routinely collected, and were retrieved from the patient file. The HMIS tools used for routine data collection include paper-based tools, such as the patient file and registers. Periodic programme reports were made using data from these registers, to monitor the project. Data were extracted on paper files and then entered in an electronic database by data clerks and coded collected data were kept in an Excel database. No names or addresses were included. Data on variables of interest was collected i.e. demographic data—including age structure of study participants, sex, occupation, educational level and duration on ART, current WHO clinical stage, retention, adherence and viral suppression.

Retention in care on ART was defined as the proportion of patients that are linked to care at 6 months ART, among those who started ART and those that were transferred out to another ART facility.

Attrition on ART was the opposite of retention in care, and was defined as the proportion of patients that were either dead, LTFU or who stopped ART, among those who started ART, and that were not transferred out.

Patients LTFU included those lost from the care continuum for more than 2 months since the last appointment. We distinguish between clients who were LTFU immediately after starting ART from those LTFU after their second ART visit, once the patient engaged in ART follow-up. Immediate LTFU identifies those who didn’t return after ART initiation (often the same day they were tested for HIV), while LTFU after the second ART visit includes all the others who either stopped or didn’t return for a next visit.

Patients who ceased to engage in the continuum of care (stopped ART) because of their own wishes or beliefs or because of barriers to continued access to care were said to have stopped ART.

Viral suppression was defined as having a viral load less than 1000 copies/ml. Good adherence to medication was defined as >90% ART pill intake. Poor adherence was defined as the opposite. Adherence was assessed using patient self-report and pill count during each clinic or outreach visit. Patients who missed more than 3 doses/month were categorized having a poor adherence to medication.

### Data analysis

The data analysis was done using Stata version 11 (College Station, TX: StataCorp LP.).

Numeric variables were analyzed using medians and interquartile ranges while calculation of proportions was used for categorical variables. Some numerical variables were categorized for statistical analysis.

Kaplan–Meier techniques was used to estimate retention over time. The Log-rank test was used to estimate differences between Kaplan-Meier curves. We employed univariate and multivariate logistic regression to estimate the association between the type of KP and immediate LTFU. Moreover, we conducted a Cox proportional hazard regression to estimate the association between attrition and sociodemographic and clinical patient characteristics. Patients who died, were LTFU, or stopped ART were considered as having experienced the event. Patients active on 31st August 2017 were censored on this date. Patients transferred out to another clinic were censored the date they were transferred out. Through backwards elimination the saturated multivariable model (including all variables) was simplified until only the variable of interest (key population type) and variables significantly associated with attrition remained. The threshold for significance was set at p<0.05. For covariates, missing observations were handled using the missing indicator method. Patients without outcome were excluded.

### Ethics statement

This research used data collected as part of routine care and treatment services for KP that were supported by the United States Agency for International Development (USAID) through a cooperative agreement with the Society for Family Health (SFH) in Nigeria. Consent was obtained from the local Research Measurement and Result department of SFH to use program data for analysis and as it was not practicable to obtain consent from patients who were retrospectively included in the study, we requested a waiver from the IRB, Institute of Tropical Medicine (ITM) Antwerp. The local IRB (SFH) approved the study while the latter IRB (ITM) also approved the study, including lack of consent. We maximally protected participants as all data were fully anonymized before accessing them: a) data in the study database did not include identifying variables, such as names or addresses, b) the study database was encoded by staff of the routine programme.

## Results

During the study period, 15274 KP and partners received HIV testing in the community, of whom 935 were diagnosed with HIV disease (6.1% HIV positivity rate). 77.4% (724) joined the activities of the outreach team and were started on ART. The proportion of KP who started ART was 75% (537/720), 68% (32/47), 53% (8/15) and 93% (143/153) for FSW, MSM, PWID and their partners, respectively.

Of 724 patients who started ART between August 2016 and February 2017, 14 (1.9%) were excluded from further analysis because of incomplete information about their treatment outcome in the patient file.

79.7% of 710 KP in the CBART programme were within the age range of 20- 40years. Median age was 30 years (IQR: 24–35). 77.3% (549) of participants were female. Of 710 KP, 74.2%, 4.5%, 1.1% and 20% were FSW, MSM, PWID, and Partners of KP, respectively (Table 1).

**Table 1:** Baseline characteristics of key-populations and their partners starting ART in a community-based HIV care programme, in Nasarawa state, Nigeria, between August 2016 and February 2017.

	FSW*		MSM^+^		PWID^$^		Partner^#^		Total	
	N	(Col%)	N	(Col%)	N	(Col%)	N	(Col%)	N	(Col%)
Total	527		32		8		143		710	
Gender (n = 710)										
Male	0	(0.0)	32	(100.0)	8	(100.0)	121	(84.6)	161	(22.7)
Female	527	(100.0)	0	(0.0)	0	(0.0)	22	(15.4)	549	(77.3)
Age (n = 710)										
<20	26	(4.9)	3	(9.4)	1	(12.5)	4	(2.8)	34	(4.8)
20-<30	246	(46.7)	24	(75.0)	5	(62.5)	39	(27.3)	314	(44.2)
30-<40	188	(35.7)	5	(15.6)	2	(25.0)	57	(39.9)	252	(35.5)
> = 40	67	(12.7)	0	(0.0)	0	(0.0)	43	(30.1)	110	(15.5)
Education (n = 655)										
Formal	375	(77.2)	29	(96.7)	4	(80.0)	115	(85.8)	523	(79.8)
Quranic	30	(6.2)	0	(0.0)	0	(0.0)	5	(3.7)	35	(5.3)
None	81	(16.7)	1	(3.3)	1	(20.0)	14	(10.4)	97	(14.8)
Unemployed (n = 657)										
No	162	(33.2)	15	(50.0)	2	(40.0)	69	(51.5)	248	(37.7
Yes	326	(66.8)	15	(50.0)	3	(60.0)	65	(48.5)	409	(62.3
Same-day ART initiation (n = 710)										
No	2	(0.4)	0	(0.0)	0	(0.0)	0	(0.0)	2	(0.3
Yes	525	(99.6)	32	(100.0)	8	(100.0)	143	(100.0)	708	(99.7
Tuberculosis before ART (n = 709)										
No TB signs	523	(99.4)	31	(96.9)	7	(87.5)	139	(97.2)	700	(98.7)
Presumptive TB	2	(0.4)	1	(3.1)	1	(12.5)	4	(2.8)	8	(1.1)
On TB treatment	1	(0.2)	0	(0.0)	0	(0.0)	0	(0.0)	1	(0.1)
WHO staging when starting ART (n = 710)										
I&II	513	(97.3)	32	(100.0)	7	(87.5)	133	(93.0)	685	(96.5)
III&IV	14	(2.7)	0	(0.0)	1	(12.5)	10	(7.0)	25	(3.5)

+- men who have sex with men

*- female sex workers

$-people who inject drugs

#-those in sexual relationship with KP

99.7% of KP started ART the same day they were tested and confirmed HIV positive (Table 1). One person started ART one day after HIV testing and counselling, a second person after 2 days. The majority of KP (96.5%) had mild HIV disease (WHO clinical stage I and II) at ART enrollment and on initiation.

14.8% of KPs were without any form of education, 5.3% had quranic form of education (non-formal) while 79.8% had a formal education. Most of the MSM were educated with 96.7% having formal education (ranging from primary to tertiary education) followed by PWID (85.8%) and FSW (77.2%) (X^2^: p = 0.09). The majority (62.3%) was unemployed: 66.8% of FSW, 50% of MSM and 60.0% of PWID were unemployed (X^2^: p = 0.001) (Table 1).

Of 454 who were active in care on 31/08/2017, 35 (7.7%) had experienced a treatment interruption but reengaged into care.

All patients had started ART 6 months or more before the end of the study period. Median follow-up time was 226.0 days (IQR: 34.0–293.0). Among FSW, MSM, PWID and partners it was 226.0 days (IQR: 31.0–306.0), 216.5 days (IQR: 28.0–262.0), 166 days (29.0–257.5) and 233 days (IQR:65.0–306.0), respectively.

87.3% of KP showed good medication adherence (missed <3 tablets/30 doses). Of the 178 clients (KP) with initial viral load test results, 87% achieved viral suppression of <1000copies/ml. 74.9% of the study population are yet to have a viral load test (Table 2).

**Table 2:** Outcomes of key-populations in a community-based HIV care programme, in Nasarawa state, Nigeria, between August 2016 and February 2017.

	FSW*		MSM^+^		PWID^$^		Partner^#^		Total	
	N	(Col%)	N	(Col%)	N	(Col%)	N	(Col%)	N	(Col%)
Total	527		32		8		143		710	
Adherence ^c^ (n = 578)										
Yes	380	(87.0)	21	(95.5)	6	(85.7)	98	(87.5)	505	(87.4)
No	57	(13.0)	1	(4.5)	1	(14.3)	14	(12.5)	73	(12.6)
VL^++^ monitoring (n = 710)										
Without VL	393	(74.6)	30	(93.8)	8	(100.0)	101	(70.6)	532	(74.9)
With VL	134	(25.4)	2	(6.3)	0	(0.0)	42	(29.4)	178	(25.1)
Viral suppression (n = 178)										
No	16	(11.9)	0	(0.0)	0	(NA)	7	(16.7)	23	(12.9)
Yes	118	(88.1)	2	(100.0)	0	(NA)	35	(83.3)	155	(87.1)
Outcomes 31/08/17 (n = 710)										
Active	332	(63.0)	21	(65.6)	4	(50.0)	97	(67.8)	454	(63.9)
Transferred out	46	(8.7)	5	(15.6)	2	(25.0)	13	(9.1)	66	(9.3)
Dead	15	(2.8)	0	(0.0)	1	(12.5)	8	(5.6)	24	(3.4)
LTFU after 1^st^ ART visit ^a^	79	(15.0)	4	(12.5)	1	(12.5)	15	(10.5)	99	(13.9)
LTFU after 2^nd^ ART visit ^b^	55	(10.4)	2	(6.3)	0	(0.0)	10	(7.0)	67	(9.4)

*- female sex worker

+-men who have sex with men

$- people who inject drugs

#- Partner–those in sexual relationship with KP

++- viral load

^a^ LTFU after 1^st^ ART visit: patients who disengaged from care immediately after ART initiation and failed to return for their appointment after starting ART

^b^ LTFU after 2nd ART visit: patients who disengaged from care after their first ART follow-up visit

^c^ Medication adherence >90% adherence to ART

On the 31th of August 2017, after a median follow-up time of 226 days (about 7 months) on ART, 73.2% (active = 63.9%; transferred out = 9.3%) of clients were retained, 23.4% were LTFU and 3.4% died. Out of 710 who started ART, 99 (13.9%) didn’t return and were LTFU or reported as having stopped treatment after the first ART visits (Table 2). After the first follow-up visit on ART, another 67 patients were LTFU. LTFU rates at 3, 6 and 9 months ART were 22.3%, 24.1% and 24.5%, respectively. Mortality rates at 3, 6 and 9 months ART were 3.4%, 3.7% and 3.9%, respectively.

Except quranic education (OR 3.3; 95% CI 1.5–7.2), none of the categories of the patient characteristics was associated with immediate discontinuation (table not shown).

**Fig 1:**
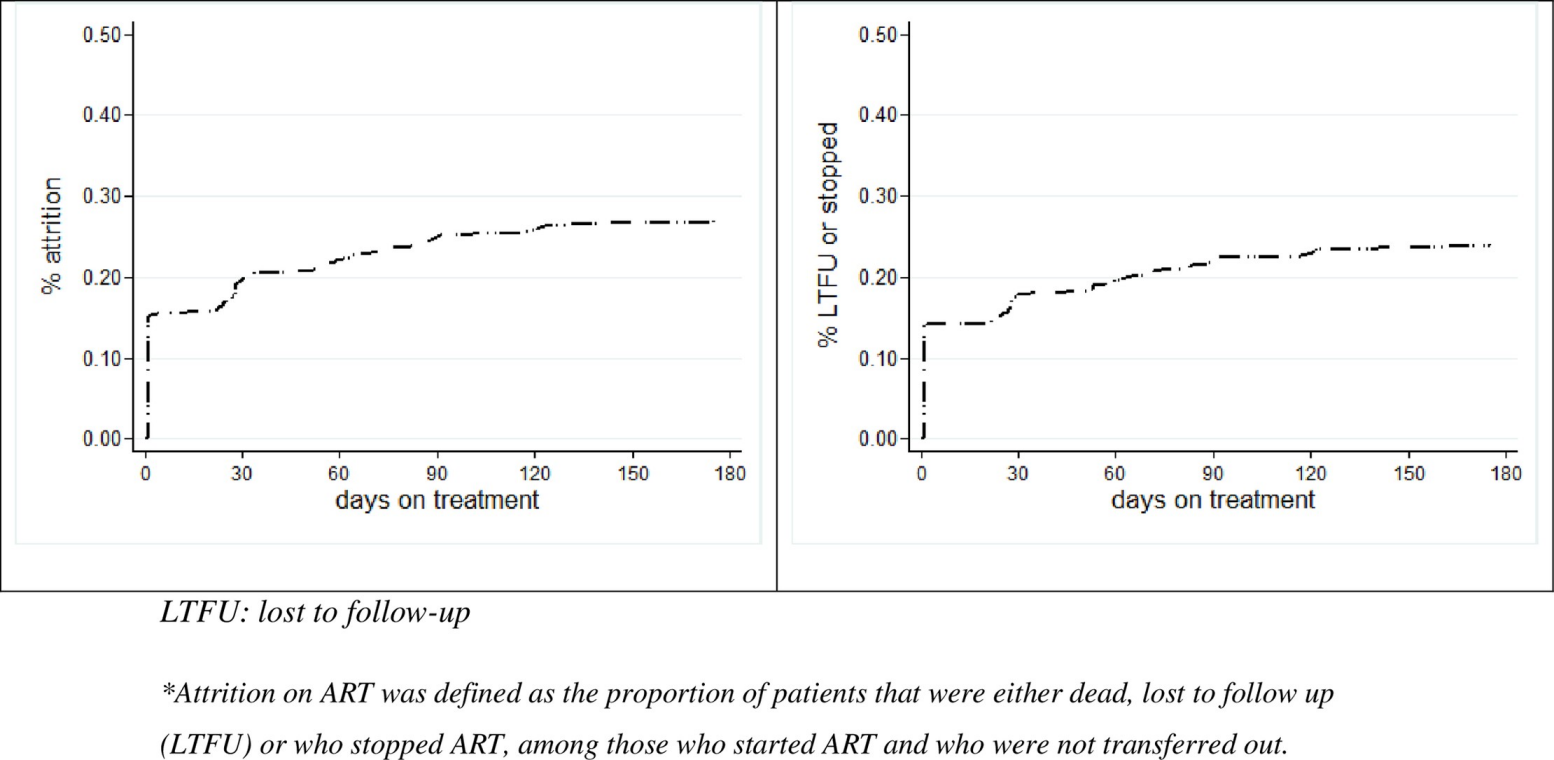
Attrition * in key populations and their partners started on ART in a community-based HIV care programme, in Nasarawa state, Nigeria, between August 2016 and February 2017.

For different KP attrition at 6 months ranged between 22.2% and 33.3%, but differences were not significant. The proportion of KP who immediately discontinued following first ART visit ranged between 10.5% and 15.0%, but differences were not significant (Fig 1).

Contact tracking identified 23 silent transfers to other facilities. Phone tracking of clinic defaulters often did not resolve the client’s treatment status, as information (mobile no./physical address) was lacking or incomplete in the patients’ folder/card, or those who answered refused to talk on the phone.

Lack of education (aHR = 1.8 (95%CI: 1.3–2.6); p = 0.001), and unemployment (aHR = 1.8 (95%CI: 1.2–2.6); p<0.001) predicted attrition. KP type, severity of HIV disease, gender and age were not significantly associated with attrition in key population on ART (Table 3).

**Table 3:** Predictors of attrition* on treatment in key populations and their partners started on ART in a community-based project, in Nasarawa state, Nigeria between August 2016 and February 2017.

	HR	(95%CI)	p	aHR	(95%CI)	p
Gender						
• Male	1					
• Female	1.3	(0.9–1.8)	0.2			
Age (n = 710)						
• <20	1.4	(0.8–2.5)	0.3			
• 20-<30	1					
• 30-<40	1.1	(0.8–1.5)	0.7			
• > = 40	1.0	(0.7–1.5)	1.0			
Education (n = 655)						
• Formal	1			1		
• Quranic	2.1	(1.2–3.6)	**0.008**	1.7	(0.95–2.9)	0.07
• None	2.2	(1.5–3.1)	**<0.001**	1.8	(1.3–2.6)	**0.001**
Unemployed (n = 657)						
• No	1			1		
• Yes	2.1	(1.5–3.0)	**<0.001**	1.8	(1.2–2.6)	**0.001**
WHO staging when starting ART (n = 710)						
• I&II	1					
• III&IV	1.7	(0.9–3.1)	0.1			
Key population type						
• Partners	1			1		
• FSW	1.3	(0.9–1.8)	0.2	1.1	(0.7–1.5)	0.8
• MSM	0.8	(0.4–2.0)	0.7	0.9	(0.4–2.1)	0.8
• PWID	1.2	(0.3–4.8)	0.8	1.0	(0.3–4.4)	0.96

fsw- female sex worker, msm-men who have sex with men, PWID- people who inject drugs

*Attrition on ART was defined as the proportion of patients that were either dead, lost to follow up (LTFU) or who stopped ART, among those who started ART and who were not transferred out.

## Discussion

Community-based HIV care has been proposed to overcome barriers to care experienced by KP, such as FSW, MSM, and PWID. However, to the best of our knowledge, a combination of community-based HIV testing and ART care has not yet been studied in KP. In Nigeria, HIV testing and ART care was provided by CBO’s, supported by an outreach team. Of 935 KP diagnosed with HIV in the community, 724 (77.4%) were started on ART in the community-based HIV care programme. Linkage to ART was much higher than what was reported previously in Nigeria. A 2015 study conducted in a similar project showed 4% linkage to ART before the implementation of community-based HIV care and 25% in a programme within 3 months of intervention [13]. Accounting for the higher linkage to ART in our study is involvement of community-based organizations, provision of incentives for complete referral and periodic mentoring/training of community facilitators by project staff.

When not contraindicated, ART was started the same day the HIV test result was confirmed. After a median follow-up time of 7 months on ART, early treatment outcomes of 710 KP and their partners on ART were determined: 73.2% of patients were retained, 87.3% adhered to medication (ARV) and 87.1% achieved viral suppression. Lack of formal education (aHR 1.8; 95% CI 1.3–2.6) and unemployment (aHR 1.8; 95% CI 1.2–2.6) were significantly associated with attrition. In our study, attrition was mainly explained by LTFU. Of 710 KP who started ART, 13.9% (99/710) discontinued after the first visit, and 23.4% (166/710) were LTFU after a median of 7 months follow-up.

Thus, the majority (99 of 166) of those LTFU discontinued immediately after ART initiation and did not return for their first follow-up visit on ART. To optimize linkage to care after a positive HIV test, the vast majority of KPs (99%) were enrolled on ART the same day they were confirmed HIV positive. A recent trial showed 80% 12-month retention after HIV testing followed by same-day ART initiation [14]. Similar findings were reported after 10 months by another trial [15]. Attrition was substantially higher in our cohort. However, findings generated from controlled facility-based trial settings with study participants selected from the general population may not be generalizable to the programmatic reality of our community-based HIV care programme for KP. Moreover, our findings showing high attrition after same-date ART initiation cannot be compared with reports of low attrition in other community-based ART programs (CBART). CBART programs usually target patients already stable on ART. One Malawian study conducted in the general population offered home-tested clients the option to start ART at home. After ART initiation, patients were linked to facility-based care. The study showed 28.7% attrition at 6 months ART, similar to the attrition reported by our study.

Few studies report on linkage between HIV testing and ART initiation among KP [16]. One Rwandan study showed that 27% of HIV-positive FSW did not return for a post diagnosis visit and thus did not start ART [17]. If in our setting ART would not have been offered the same day that patients were diagnosed to be HIV-positive, likely early attrition would have been less high. We speculate that poor treatment preparation in terms of adherence might account for the initial high rate of attrition on ART. Furthermore, high mobility of KP may explain attrition. This is especially true for commercial sex workers who frequently change their base in search of greener pasture and for fear of HIV status discovery by others, thereby reducing their customer base. Moreover, due to the discriminatory stance of the Nigerian community on MSM and the government position on MSM activities, MSM tend to travel a lot in a bid to hide their identity. Similarly, in Ethiopia, it was reported that sex workers were highly mobile, moving in order to attract a wider or different client base, for adventure, and to conceal illnesses which might be associated with AIDS [18].

Reported mortality was low, 3.4% after a median follow-up time of 7 months on ART. Moreover, tracing of patients LTFU showed that some self-transferred to another ART site. In our study setting ART is decentralized and accessible. Likely many patients that were LTFU in our study reengaged in care later on, for example when experiencing a new health condition that required clinical follow-up or even an admission. One recent South African study showed that mortality among patients was low when ART was accessible in an urban community characterized by unemployment and poverty [19].

Only a minority of patients accessed viral load monitoring. Nevertheless, 87.1% viral suppression within 6 months after starting ART, compares favorably with 79.4% reported by a study among FSW in Burkina Faso [20]. However, viral load coverage was low, and limited the interpretation of viral suppression in this cohort. Identified barriers to viral load testing among KP were poor logistics for viral load consumables, difficulties with assuring a cold chain for viral load sample transfer from outreach venues to the PCR-lab and a long turn-around time between sample collection and obtaining results at the outreach venue. Furthermore, a significant number of KP were LTFU before they were eligible for their first viral load testing at 6 months on ART. To optimize viral load testing among KP, both retention and lab procedures should improve.

Few studies on CBART assessed socio-economic predictors. A study conducted in Kinshasa, DRC, among stable HIV patients in community based ART centre, showed that sociodemographic factors, such as gender, marital status and educational level, were not associated with attrition. Hence, the authors concluded that the model was suitable for heterogenous patient groups [21]. However, our study revealed that lack of formal education and unemployment were strongly associated with attrition among the KP and their partners. Structural barriers to ART care, such as poverty and a low level of literacy were identified long ago [22]. CBART programs may need to consider to liaise with other CBO’s, which may complement health interventions with socio-economical interventions. Care provision within and for communities will only be sustained on the long term when a holistic approach is employed, and when community stakeholders join the prioritization and planning of activities [23].

The community-based ART model in the Nigerian state leveraged on community-based organizations to provide ART services to KP. Within the context of community-based ART model on the project, community based organizations engaged KP through their peers for improved linkage, medication adherence, retention and viral suppression. Indeed, engagement of NGOs/CBOs for specific KP in Cameroun suggest that these organizations can provide entry into the continuum of care through tailored peer outreach, prevention and supportive services for lesbian, gay, bisexual and transgender communities. MSM in Douala, Cameroun were more likely to have accessed NGO/ CBO services or reached by an outreach worker if they were living with HIV (aOR 3.60 CI 1.35–9.60. p = 0.01). This study recommended scale-up of community-led HIV interventions using social networks to increase health service uptake, improve health outcomes and decrease onward transmission of HIV [11]. In Uganda, community health extension workers linked 64% of PLHIVs to care in a bid to expand HIV testing and linkage in the community [24].

This study has several strengths. First, this study reflects the reality of a comprehensive community-based HIV care program for KP. Data were derived from routine program tools used for monitoring and evaluation. Moreover, data collection and verification was done rigorously, triangulating data from multiple data sources, such as patient files and registers. However, the study has limitations as well. Data for some variables were incomplete in the service registers and ART files. Fourteen patients were excluded because no outcome was available. The study period was short. Additional studies are needed to determine long-term treatment outcomes among KP that are living with HIV. Moreover, this study was conducted in a single setting. Implementation across the country and in other countries may inform if our findings can be transferrable to other settings. Finally, the reasons for immediate discontinuation from program need to be explored, as wells as how counselling could explore better psychosocial readiness to start ART.

In conclusion, this study has shown that it is feasible to deliver a comprehensive package including HIV testing and ART care within the community. This community-based HIV care model for KP resulted in a high uptake of HIV prevention and treatment services in our study setting. However, ART initiation on the same day of confirmatory HIV testing likely inflated attrition during the early follow-up period on ART. We speculate that early attrition among KP after same-day ART can be mitigated by assessing better the psychosocial readiness of clients.
